# Exercise Made Accessible: the Merits of Community-Based Programs for Persons with Parkinson’s Disease

**DOI:** 10.1007/s11910-023-01303-0

**Published:** 2023-10-04

**Authors:** Anneli Langbroek-Amersfoort, Sabine Schootemeijer, Lars Bouten, Bastiaan R. Bloem, Nienke M. De Vries

**Affiliations:** Center of Expertise for Parkinson & Movement Disorders, Department of Neurology, Donders Institute for Brain, Cognition and Behavior, Radboud University Medical Center, Nijmegen, The Netherlands

**Keywords:** Parkinson’s disease, Community-based exercise, Sport, Physiotherapy

## Abstract

**Purpose of Review:**

Many studies have identified positive effects of physiotherapy and exercise for persons with Parkinson’s disease (PD). Most work has thus far focused on the therapeutic modality of exercise as used within physiotherapy programs. Stimulated by these positive findings, there is now a strong move to take exercise out of the clinical setting and to deliver the interventions in the community. Although the goals and effects of many such community-based exercise programs overlap with those of physiotherapy, it has also become more clear that both exercise modalities also differ in various ways. Here, we aim to comprehensively review the evidence for community-based exercise in PD.

**Recent Findings:**

Many different types of community-based exercise for people with PD are emerging and they are increasingly being studied. There is a great heterogeneity considering the types of exercise, study designs, and outcome measures used in research on this subject. While this review is positive regarding the feasibility and potential effects of community-based exercise, it is also evident that the general quality of these studies needs improvement.

**Summary:**

By focusing on community-based exercise, we hope to generate more knowledge on the effects of a wide range of different exercise modalities that can be beneficial for people with PD. This knowledge may help people with PD to select the type and setting of exercise activity that matches best with their personal abilities and preferences. As such, these insights will contribute to an improved self-management of PD.

**Supplementary Information:**

The online version contains supplementary material available at 10.1007/s11910-023-01303-0.

## Introduction

Many studies have reported on the positive effects of both physiotherapy and exercise in persons with Parkinson’s disease (PD) [1, 2]. Frequently, no clear distinction is being made between these two options. In previous guidelines, reviews, and meta-analyses on physiotherapy, exercise was included equally to other therapeutic modalities (i.e., balance training, strategy training) [1–4]. In the past years, however, the evidence for many different types of community-based exercise for people with PD has increased enormously. Although the goals and effects of many such community-based exercise programs overlap with those of physiotherapy, it has become more clear that both modalities also differ in various ways.

It is interesting to consider some differences between exercise as part of physiotherapy versus community-based exercise programs. Physiotherapy works according to movement-related questions for help, SMART (specific, measurable, achievable, relevant, and time-bound) treatment goals, and a personalized treatment plan. In contrast, community-based exercise can be performed just for the fun of it [5]. Physiotherapists can use exercise as a treatment modality during their therapy, but they are usually not trained as instructors in specific types of exercise, such as boxing or dance. On the other hand, exercise instructors are normally not specialized in the consequences of having PD and the specific challenges that these may generate when engaging in an exercise program. For example, people with PD may respond differently on high-intensity exercise because of autonomic dysfunction [6]. Just like exercise programs led by trained physiotherapists, community-based exercise may also have positive effects on general health, on PD symptoms, and possibly even on disease progression [7]. However, these positive effects are, unlike physiotherapy, typically not quantified during training. Moreover, community-based exercise is usually not adjusted to the personal goals or individual capacities of each person with PD, yet both of these may be affected because of the impact of PD. This raises safety concerns when instructors do not have specialized knowledge of PD. On the other hand, performing exercise in the community offers many different options and practical advantages: it is often fun and includes a social element when performed in groups, it is performed close to home (thus reducing traveling time) and is generally much more accessible, and people can start on their own initiative and decide for themselves where, when, and how often they participate. Community-based exercise is therefore an important tool for self-management in people with PD [8].

Because of these crucial differences between physiotherapy and community-based exercise, we propose to make a clearer distinction between these two approaches, both in research and clinical practice. A personalized assessment is needed to decide whether community-based exercise is sufficient, or whether additional specialized physiotherapy is needed. We also need to further consider the level of PD-specific knowledge and expertise that is needed for instructors to work with people with PD. Finally, more research on the cost-effectiveness and dosing is necessary. A previous review indicated that community-based exercise may improve motor functions [9]. However, only motor functioning was considered as outcome in this study. We here aim to comprehensively review the evidence of different types of community-based exercise in PD. We deliberately chose to exclude aerobic exercise and strength training from this review while these can also be performed as community-based exercise in for example the gym. We excluded these types of exercise because [1] these training modalities are often also performed in physiotherapy (i.e., physiotherapists are trained in using these training modalities and they are part of the standard physiotherapy repertoire) and [2] both, but in particular aerobic exercise, have already been extensively studied and reviewed, indicating that aerobic exercise has a positive effect on cardiorespiratory fitness and motor symptoms [10–13]. Importantly, a disease-modifying potential is hypothesized based on animal studies [14, 15], observational studies [16, 17], and the first observations in clinical trials in humans [18, 19]. Future clinical trials will need to confirm and unravel this disease-modifying potential. Considering strength training, we know that this positively impacts muscle strength, motor problems, mobility, and balance [20, 21].

By focusing on community-based exercise, we hope to generate more knowledge on the effects of a wide range of different exercise modalities that can be beneficial for people with PD. This knowledge may help people with PD to select the type and setting of exercise activity that matches best with their personal abilities and preferences. As such, these insights will contribute to an improved self-management of PD.

## Methods

We reviewed papers that evaluated the effects of community-based exercise in people with PD. We searched PubMed between January 2013 and November 2022 as part of the development of a new guideline for allied healthcare professionals in PD in The Netherlands (Supplementary 1). This search resulted in many hits for articles on community-based exercise, which were not included in the guideline because we classified them as not being physiotherapy. To not let this comprehensive overview go to waste, we decided to perform additional search specifically aimed at community-based exercise and review the current literature on this topic. We included randomized controlled trials (RCT) and controlled clinical trials (CCT) that compared the effect of any type of community-based exercise with (any type of) control intervention and included people with PD.

### Study selection

Two reviewers screened all papers independently for inclusion. Title and abstract were screened and when necessary the full text was retrieved and evaluated. Disagreements were solved in a discussion meeting and all inclusions were based on consensus between the reviewers.

### Data extraction

We extracted data from all articles using a standardized data collection form. We extracted the following data: (1) publication information (author, year), (2) study design, (3) study sample (size, age, in- and exclusion criteria), (4) intervention (type, frequency, duration, supervision, location), (5) primary and secondary outcomes, (6) results (primary and secondary outcomes), (7) conclusions. We categorized the community-based exercise into (1) yoga, (2) wuqinxi and qigong, (3) tai chi and ai chi, (4) dance, (5) Pilates, (6) (Nordic) walking, (7) climbing, and (8) kayaking.

### Outcome measures

Because of the large heterogeneity in outcomes used, we decided not to limit this review based on specific outcomes. However, we did focus primarily on the primary outcomes as reported in the included papers and on the between group differences at follow-up in Table 1. In order to be as comprehensive as possible, we report the secondary outcomes in the Supplementary table.

**Table 1:** Primary outcomes as reported in the included papers

Authors (year)	Design	Sample	Primary outcome	Intervention	Control group	Result primary outcome
Yoga (total *n* = 275)						
Ni et al. (2016) [22]**	Secondary analysis	*N*: 27 Age: 60–90 yrs HY: I–III MMSE: ≥ 24	NA	Power yoga, 2 × /wk, 1 h/ × , 12 wks, supervised, group	Usual care with health education, 1 × /month, 12 wks	NA
Kwok et al. (2019) [23]	Single-blinded RCT	*N*: 138 Age: > 18 yrs HY: I–III AMTS: < 6	HADS	Mindfulness yoga, 1 × /wk, 1.5 h/ × , 8 wks, supervised, group AND Mindfulness yoga, 2 × /wk, 20 min/ × , 8 wks unsupervised, individual	Stretching and resistance training, 1 × /wk, 1 h/ × , 8 wks, supervised, groups. 2 × /wk 20 min/ × , 8 wks unsupervised, individual	HADS: yoga >> CON
Van Puymbroeck et al. (2018) [24]	RCT	*N*: 30 Age: ≥ 18 yrs HY: I1/2–III Short minimental status exam: ≥ 4	NA	Yoga, 2 × /wk, 1 h/ × , 8 wks, supervision, group	Wait list	NA
Cherup et al. (2021) [25]	RCT	*N*: 46 Age: 40–90 yrs HY: I–III Cognition: NA	Joint position sense, joint kinesthesia, Tinetti balance assessment	Yoga meditation, 2 × /wk, 45 min/ × , 12 wks, supervised, group	Proprioceptive training, 2 × /wk, 45 min/ × , 12 wks, supervised, group	Joint position sense: 0 Joint kinesthesia: yoga >> CON Tinetti balance assessment: yoga >> CON
Walter et al. (2019) [26]**	RCT	*N*: 30 Age: NA yrs HY: I1/2 SIS: ≥ 4	PFS-16, ABC, FCS, FMS, ACS, PDQ-8	Yoga, 2 × /wk, 1 h/ × , 8 wks, supervised, group	Wait list	PFS-16: yoga << control ABC: yoga >> control FCS: yoga >> control FMS: yoga >> control ACS: yoga >> control PDQ-8: yoga << control
Kwok et al. (2022) [27]**	Multicenter RCT	*N*: 138 Age: > 18 yrs HY: I–III AMTS: ≥ 6	NA	Mindfulness yoga, 1 × /wk, 1.5 h/ × , 8 wks, supervised, group	Strength and resistance training, 1 × /wk, 1 h/ × , 8 wks, supervised, group	NA
Elangovan et al. (2020) [28]	RCT (pilot)	*N*: 20 Age: 45–75 yrs HY: I–III MoCA: ≥ 26	NA	Hatha yoga, 2 × /wk, 1 h/ × 12 wks, group/personal unclear, supervised	Wait list	NA
Ni et al. (2016) [22]	RCT	*N*: 41 Age: 60–90 yrs HY: I–III MMSE: ≥ 24	MDS-UPDRS III	Power training, 2 × /wk, 3 circuits of 10–12 reps, 12 wks, personal, supervised AND Yoga, 2 × /wk, 1 h/ × , 12 wks, group, supervised	Health education class, non-exercise	MDS-UPDRS III: PWT: + ; yoga: + ; CON: 0
Wuqinxi and qigong (total *n* = 477)						
Wang et al. (2020) [29]	RCT	*N*: 46 Age: 55–80 yrs HY: I–III Cognition: cognitive impairment based on medical history and/or clinical assessment	NA	Wuqinxi, 2 × /wk, 1 h/ × , 12 wks, supervised, group/personal unclear	Stretching, 2 × /wk, 1 h/ × , 12 wks, supervised, group/personal unclear	NA
Shen et al. (2021) [30]	RCT	*N*: 32 Age: 55–80 yrs HY: I–III MMSE: ≥ 24	Stroop color and word test/FAB/MoCA	Wuqinxi, 2 × /wk, 1.5 h/ × , 12 wks, supervised, group/personal unclear	Stretching, 2 × /wk, 1.5 h/ × , 12 wks, supervised, group	ST1: wuqinxi >> stretching; wuqinxi: + ; stretching: + ST2: wuqinxi = stretching; wuqinxi: 0; stretching: 0 FAB: wuqinxi >> stretching; wuqinxi: + ; stretching: + MoCA: wuqinxi >> stretching; wuqinxi: + ; stretching: +
Wan et al. (2021) [31]	RCT	*N*: 52 Age: 40–85 yrs HY: I–IV MMSE: ≥ 24	NA	Qigong, 4 × /wk, 1 h/ × , 12 wks, supervised, group/personal unclear	Routine stable drug treatment was maintained within 12 weeks without any other intervention	NA
Xiao and Zhuang (2016) [32]	Single-blinded RCT	*N*: 100 Age: 55–80 yrs HY: I–III MMSE: ≥ 23	UPDRS, Parkinson’s Disease Sleep Scale-2, Parkinson Fatigue Scale (PFS-16), MMSE, BBS, TUG, 6 camera Vicon 512 motion capture system (to test gait), Freezing of Gait questionnaire	Part 1: Baduanjin qigong, 4 × 45 min supervised, group/personal unclear; audiovisual learning package of 12–15 min (8 exercises repeated 6 times at 43–49% of max HR) unsupervised Part 2: Baduanjin qigong, 4 × /week, 1 × per day, 6 months AND walking, 7 × /week, 0.5 h/ × , 6 months, unsupervised	Walking, 7 × /week, 0.5 h/ × , 6 months, unsupervised	UPDRS: qigong: + ; CON: 0 PDSS-2: qigong: + ; CON: 0 BBS: qigong: + ; CON: 0 6 MW: qigong: + ; CON: 0 TUG: qigong: + ; CON: 0 Gait speed: qigong: + ; CON: 0
Liu et al. (2016) [33]	RCT	*N*: 54 Age: NA yrs HY: NA Cognition: NA	Myometry (Myoton-3), BDW-85-II	Health qigong, 5 × /wk, 1 h/ × , 10 wks, supervision unclear, group/personal unclear AND Drug therapy and participation in regular daily activities	Drug therapy and participated in regular daily activities	Myometry (left): qigong: + ; CON: 0 Myometry (right): qigong: + ; CON: 0
Moon et al. (2020) [34]	RCT (pilot)	*N*: 32 Age: 40–80 yrs HY: NA MMSE: ≥ 24	NA	Qigong, 1 × /wk, 0.75–1 h/ × , 12 wks, supervised, group AND Qigong, 14 × /wk, 0.25 h/ × , 12 weeks, unsupervised, personal	Sham qigong, 1 × /wk, 0.75–1 h/ × , 12 wks, supervised, group AND Sham qigong, 14 × /wk, 0.25 h/ × , 12 weeks, unsupervised, personal	NA
Li et al. (2022) [35]	Single-blinded RCT	*N*: 40 Age: 60–80 yrs HY: I–III MMSE: ≥ 24	Gait parameters measured on a walkway: - Single-task - Obstacle crossing - Backward digit span - Serial-3 subtraction	Wuqinxi qigong, 2 × /wk, 1.5 h/ × , 12 wks, supervised, group	Stretching, 2 × /wk, 1.5 h/ × , 12 wks, supervised, group	Single-task (gait speed): wuqinxi qigong (WQ): + ; CON: 0; WQ >> CON Single-task (stride length): WQ: + ; CON: 0; WQ >> CON Single-task (double support): WQ: 0; CON: + ; WQ = CON Obstacle crossing (gait speed): WQ: 0; CON: 0; WQ << CON Obstacle crossing (stride length): WQ: 0; CON: 0; WQ = CON Obstacle crossing (double support): WQ: + ; CON: 0; WQ >> CON Backward digit span (gait speed): WQ: 0; CON: 0; WQ = CON Backward digit span (stride length): WQ: 0; CON: 0; WQ = CON Backward digit span (double support): WQ: 0; CON: 0; WQ >> CON Serial-3 subtraction (gait speed): WQ: 0; CON: 0; WQ = CON Serial-3 subtraction (stride length): WQ: + ; CON: 0; WQ = CON Serial-3 subtraction (double support): WQ: 0; CON: 0; WQ >> CON
Li et al. (2022) [35]	RCT	*N*: 40 Age: 50–80 HY: I–III Cognition: NA	NA	Health qigong, 5 × /wk, 1 h/ × , 12 wks, supervised	Wait list	NA
Wang et al. (2022) [36]	RCT	*N*: 60 Age: 50–80 HY: I–II Cognition: NA	NA	Wu Qin Xi, 3 × /wk, 90 min/ × , 24 wks, supervised, group	Stretching exercise, 3 × /wk, 90 min/ × , 24 wks, supervised, group	NA
Amano et al. (2013) [37]*	Independent single-blinded randomized controlled trials	*N*: 21 Age: NA yrs HY: NA MMSE: > 26	(1) The magnitude of posterior and lateral COP displacement [2] The mean COP velocity in posterior and lateral directions prior to an initial heel-off of the swing limb	Qigong meditation control, 2 × /wk, 1 h/ × , 16 wks, supervised, group	Tai chi, 2 × /wk, 1 h/ × , 16 wks, supervised, group	S1DisAP: qigong = CON S1DisML: qigong << CON S1VelAP: qigong = CON S1VelML: qigong = CON
Tai chi and ai chi (total *n* = 515)						
Amano et al. (2013) [37]*	Independent single-blinded randomized controlled trials	*N*: 21 Age: NA yrs HY: NA MMSE: > 26	(1) The magnitude of posterior and lateral COP displacement (2) The mean COP velocity in posterior and lateral directions prior to an initial heel-off of the swing limb	Tai chi, 2 × /wk, 1 h/ × , 16 wks, supervised, group	Qigong meditation control, 2 × /wk, 1 h/ × , 16 wks, supervised, group	S1DisAP: tai chi = CON S1DisML: tai chi >> CON S1VelAP: tai chi = CON S1VelML: tai chi = CON
Amano et al. (2013) [37]*	Independent single-blinded randomized controlled trials	*N*: 24 Age: NA yrs HY: NA MMSE: > 26	(1) The magnitude of posterior and lateral COP displacement (2) The mean COP velocity in posterior and lateral directions prior to an initial heel-off of the swing limb UPDRS-III	Tai chi, 3 × /wk, 1 h/ × , 16 wks, supervised, group	Non-contact control	S1DisAP: tai chi = CON S1DisML: tai chi = CON S1VelAP: tai chi = CON S1VelML: tai chi = CON
Li et al. (2014) [38]	Single-blinded randomized controlled trial	*N*: 195 Age: 40–85 yrs HY: I–IV	NA	Tai chi, 2 × /wk, 1 h/ × , 26 wks, supervised, group OR Resistance training, 2 × /wk, 1 h/ × , 26 wks, supervised, group	Stretching, 2 × /wk, 1 h/ × , 26 wks, supervised, group	NA
Gao et al. (2014) [39]	Single-blinded randomized control trial	*N*: 80 Age: > 40 yrs HY: NA MMSE: ≥ 24	UPDRS III, BBS, TUG	Tai chi, 3 × /wk, 1 h/ × , 12 wks, supervised, group	No intervention	UPDRS-III: tai chi = CON BBS: tai chi >> CON TUG: tai chi = CON
Kurt et al. (2018) [40]	RCT	*N*: 40 Age: NA yrs HY: II–III MMSE: ≥ 24	Biodex-3.1, BBS, TUG, PDQ-39, UPDRS-III	Ai chi, 5 × /wk, 1 h/ × , 5 wks, supervised, group/personal unclear	Land-based exercise, 5 × /wk, 1 h/ × , 5 wks, supervision unclear, group/personal unclear	Biodex-3.1 (API): ai chi: + ; CON: + ; ai chi >> CON Biodex-3.1 (ML): ai chi: + ; CON: + ; ai chi >> CON Biodex-3.1 (OBI): ai chi: + ; CON: + ; ai chi >> CON BBS: ai chi: + ; CON: + ; ai chi >> CON TUG: ai chi: + ; CON: + ; ai chi >> CON UPDRS-III: ai chi: + ; CON: + ; ai chi >> CON PDQ-39: ai chi: + ; CON: + ; ai chi >> CON
Pérez de la Cruz (2017) [41]	Prospective single-blind RCT	*N*: 30 Age: > 40 yrs HY: I–III MMSE: ≥ 24	Visual Analog Scale (VAS) for pain	Ai chi, 2 × /wk, 45 min/ × , 10 wks, supervised, group	Dry land therapy, 2 × /wk, 45 min/ × , 10 wks, supervised, group	VAS: ai chi: + ; CON: + (ai chi more significant)
Pérez de la Cruz (2018) [42]	RCT (pilot)	*N*: 29 Age: > 40 yrs HY: I–III MMSE: ≥ 24	NA	Ai chi, 2 × /wk, 45 min/ × , 11 wks, supervised, group	Dry land therapy, 2 × /wk, 45 min/ × , 11 wks, supervised, group	NA
Pérez de la Cruz (2019) [43]**	Single-blind RCT	*N*: 30 Age: > 40 yrs HY: I–III MMSE: ≥ 24	NA	Ai chi, 2 × /wk, 45 min/ × , 10 wks, supervised, group	Dry land therapy, 2 × /wk, 45 min/ × , 10 wks, supervised, group	NA
Khuzema et al. (2020) [44]*	RCT	*N*: 27 Age: 60–85 yrs HY: II1/2–III Cognition: NA	NA	Tai chi, 5 × /wk, 30–40 min/ × , 10 wks, supervised (first time) remainder of time unsupervised, personal OR Yoga, 5 × /wk, 30–40 min/ × , 10 wks, supervised (first time) remainder of time unsupervised, personal	Conventional balance exercise program, 5 × /wk, 40–45 min/ × , 10 wks, supervised (first time) remainder of time unsupervised, personal	NA
Zhang et al. (2015) [45]	RCT	*N*: 40 Age: NA HY: I–IV MMSE: ≥ 24 MMSE: ≥ 17 (if participant did not attend school) MMSE: ≥ 20 (if participant only finished elementary education)	Berg Balance Scale (BBS)	Tai chi, 2 × /wk, 60 min/ × , 12 wks, supervised, group	Multimodal exercise training, 2 × /wk, 60 min/ × , 12 wks, supervised, group	BBS: tai chi: 0; CON: + ; tai chi = CON
Poier et al. (2019) [46]*	RCT (pilot)	*N*: 29 Age: 50–90 yrs HY: NA Cognition: NA	PDQ-39	Tai chi, 1 × /wk, 60 min/ × , 10wks, supervised, group, with own partner	Tango Argentino, 1 × /wk, 60 min/ × , 10wks, supervised, group, with own partner	PDQ-39: tai chi: 0; CON: 0; tai chi = CON
Dance (total *n* = 692)						
Duncan and Earhart (2014) [47]	RCT (pilot)	*N*: 10 Age: > 40 yrs HY: NA Cognition: NA	MDS-UPDRS III	Argentine tango, 2 × /wk, 1 h/ × , 2 yrs, supervised, group	No prescribed exercise, instructed to maintain current level of physical activity	MDS-UPDRS III: tango >> CON
Rios Romenets et al. (2015) [48]	RCT (pilot)	*N*: 33 Age: NA yrs HY: I–III Cognition: no dementia	MDS-UPRDS-3	Argentine tango, 2 × /wk, 1 h/ × , 12 wks, supervised, group	Wait list	MDS-UPDRS III: tango = CON
Hashimoto et al. (2015) [49]	Quasi RCT (pilot)	*N*: 59 Age: NA yrs HY: NA Cognition: NA	NA	Dance, 1 × /wk, 1 h/ × , 12 wks, supervised, group/personal unclear OR PD-exercise, 1 × /wk, 1 h/ × , 12 wks, supervised, group/personal unclear	Usual care	
Shanahan et al. (2017) [50]	RCT (pilot)	*N*: 90 Age: NA yrs HY: I–II1/2 Cognition: NA	Feasibility: correct randomization and allocation, resources, recruitment rates, willingness of participation, attrition rates	Dancing, 1 × /wk, 1.5 h/ × , 10 wks, supervised, group AND 20-min home dance program, 3 × /wk	Usual care	Attendance was 93.5%. Compliance with home program was 71.46%
Hulbert et al. (2017) [51]	RCT	*N*: 27 Age: NA yrs HY: I–III Cognition: NA	NA	Dance (ballroom and Latin American), 2 × /wk, 1 h/ × , 10 wks, supervised, group	Usual care	NA
Lee et al. (2018) [52]	RCT	*N*: 32 Age: 50–80 yrs HY: I–III MMSE: > 20	MDS-UPDRS	Turo (qi dance), 2 × /wk, 1 h/ × , 8 wks, supervised, group/personal unclear	Wait list	UPDRS (mentation and mood): Turo = CON UPDRS (activities): Turo >> CON UPDRS (motor examination): Turo >> CON UPDRS (total): Turo >> CON
Michels et al. (2018) [53]	RCT	*N*: 13 Age: > 18 yrs HY: I–V MMSE: ≥ 24	Class attendance, safety and satisfaction	Dance/movement therapy (DT), 1 × /wk, 1 h/ × , 10 wks, supervised, group	Talk therapy/support group, 1 × /wk, 1 h/ × , 10 wks, supervised, group	No adverse events, 7 out of 9 participants in intervention group and 4 out of 4 in control group were attended 70% of the sessions (which was the aim). Satisfaction; 7 out of 9 felt they benefited, 2 felt neutral. One person did not enjoy of feel benefited from the classes, but this person was in the control group and indicated to be disappointed not to be in the DT group
Rawson et al. (2019) [54]	RCT	*N*: 119 Age: ≥ 30 yrs HY: I–IV Cognition: NA	NA	Tango, 2 × /wk, 1 h/ × , 12 wks, supervised, group OR Treadmill, 2 × /wk, 1 h/ × , 12 wks, supervised, group	Stretching, 2 × /wk, 1 h/ × , 12 wks, supervised, group	NA
Kalyani et al. (2019) [55]	Quasi-experimental parallel group pre-test post-test study	*N*: 38 Age: 40–85 yrs HY: I–III ACE: > 82	NA	Dance, 2 × /wk, 1 h/ × , 12 wks, supervised, group	Usual care	NA
Tillmann et al. (2020) [56]	Non-randomized CT (feasibility study)	*N*: 20 Age: ≥ 50 yrs HY: I–IV MMSE: ≥ 13 for illiterate people ≥ 18 for average education ≥ 26 for high schooling	NA	Brazilian samba, 2 × /wk, 1 h/ × , 12 wks, supervised, group	Monthly lectures on health, prevention of falls and psychological care, advise to avoid beginning new physical activity	NA
Frisaldi et al. (2021) [57]	Single-blind RCT (pilot)	*N*: 38 Age: NA yrs HY: I–II Cognition: NA	MDS-UPDRS III	Dance (DArT), 3 × /wk, 1 h/ × , 5 wks, supervised, group Followed by (after 30 min break): Conventional physiotherapy, 3 × /wk, 1 h/ × , 5 wks, supervised, group	Conventional physiotherapy, 3 × /wk, 1 h/ × , 5 wks, supervised, group Followed by (after 30 min break): Conventional physiotherapy, 3 × /wk, 1 h/ × , 5 wks, supervised, group	MDS-UPDRS III (total): Dance >> CON MDS-UPDRS III (upper): Dance >> CON MDS-UPDRS III (lower): Dance = CON MDS-UPDRS III (axial): Dance = CON
Foster et al. (2013) [58]	RCT	*N*: 62 Age: NA yrs HY: I–IV Cognition: NA	ACS2	Argentine tango, 2 × /wk, 1 h/ × , 52 wks, supervised, group	Usual care	Total current participation in the tango group was higher at 3, 6, and 12 months compared with baseline (Ps .008), while the control group did not change (Ps!.11). Total activity retention (since onset of PD) in the tango group increased from 77 to 90% (PZ.006) over the course of the study, whereas the control group remained around 80% (PZ.60). These patterns were similar in the separate activity domains. The tango group gained a significant number of new social activities (PZ.003), but the control group did not (PZ.71)
Kunkel et al. (2017) [59]	RCT (feasibility)	*N*: 51 Age: NA yrs HY: I–III Cognition: NA	BBS, spinal mouse	Dance, 2 × /wk, 1 h/ × , 10 wks, supervised, group	Usual care	BBS: dance = CON Spinal mouse: dance = CON
Poier et al. (2019) [46]*	RCT (pilot)	*N*: 29 Age: 50–90 HY: NA Cognition: NA	PDQ-39	Tango Argentino, 1 × /wk, 60 min/ × , 10wks, supervised, group, with own partner	Tai chi, 1 × /wk, 60 min/ × , 10wks, supervised, group, with own partner	PDQ-39: tango: 0; CON: 0; tango = CON
Solla et al. (2019) [60]	RCT (pilot)	*N*: 20 Age: NA HY: ≤ 3 MMSE: ≥ 24	NA	Ballu Sardu (Sardinian folk dance), 2 × /wk, 90 min/ × , 12 wks, supervised, group	Usual care	NA
Li et al. (2022) [35]	RCT (three arm)	*N*: 51 Age: 40–85 HY: 1–3 Cognition: NA	NA	Yang-ge dancing 5 × /wk, 60 min/ × , 4 wks, ?	CON (I) Conventional exercise 5 × /wk, 60 min/ × , 4 wks, ? CON (II) Conventional exercise plus music 5 × /wk, 60 min/ × , 4 wks, ?	NA
Pilates (total *n* = 91)						
Maciel et al. (2020) [61]	Prospective open label non-randomized controlled clinical trial study	*N*: 42 Age: > 40 yrs HY: < 3 Cognition: NA	Balance improvement, not further specified	Pilates, 2 × /wk, 1 h/ × , 6 wks, supervised, group	Regular exercise, at least 2 × /wk, NA hr/ × , 6 wks, supervised, personal	NA
Mollineda-Cardalda et al. (2018) [62]	RCT	*N*: 26 Age: NA yrs HY: I–III Cognition: no clinical history of dementia	NA	Pilates, 2 × /wk, 1 h/ × , 12 wks, supervised, group	Physical activity program (calisthenics), 2 × /wk, 1 h/ × , 12 wks, supervised, group	NA
Göz et al. (2021) [63]	RCT (pilot)	*N*: 23 Age: ≥ 18 HY: ≤ 2 MMSE: ≥ 24	NA	Pilates, 2 × /wk, 1 h/ × , 6 wks, supervised, group OR Pilates + elastic taping, 2 × /wk, 1 h/ × , 6 wks, supervised, group	Wait list	NA
(Nordic) walking (total *n* = 220)						
Cugusi et al. (2015) [64]	RCT	*N*: 20 Age: 40–80 yrs HY: I–III MMSE: ≥ 24	NA	Nordic walking, 2 × /wk, 1 h/ × , 12 wks, supervised, group	Conventional care	NA
Monteiro et al. (2017) [65]	RCT	*N*: 33 Age: > 50 yrs HY: I–IV Cognition: NA	NA	Nordic walking, 2 × /wk, 35–60 min/ × , 9 wks, supervised, group (6 familiarization sessions (35–50 min), 12 training sessions (1 h))	Free walking, 2 × /wk, 35–50 min/ × , 9 wks, supervised, group (6 familiarization sessions (35–50 min), 12 training sessions (1 h))	NA
Bang and Shin (2017) [66]	RCT (pilot)	*N*: 20 Age: NA yrs HY: I–III MMSE: ≥ 24	NA	Nordic walking, 5 × /wk, 1 h/ × , 4 wks, supervised, group/personal unclear	Treadmill, 5 × /wk, 1 h/ × , 4 wks, supervised, group/personal unclear	NA
Granziera et al. (2021) [67]	RCT	*N*: 37 Age: NA yrs HY: II–III MMSE: ≥ 24	UPDRS-III	Nordic walking, 2 × /wk, 1.25 h/ × , 8 wks, supervised, group	Walking, 2 × /wk, 1.25 h/ × , 8 wks, supervised, group	UPDRS-III: NW = CON
Mak and Wong (2021) [68]	RCT	*N*: 70 Age: > 30 yrs HY: NA MoCA: ≥ 25	MDS-UPDRS-III	Brisk walking, 3 × /wk, 1–1.5 h/ × , 26 wks, supervised, group; first 6 weeks 1 training session of 90 min and 2 self-practice sessions of 60–90 min	Upper limb and hand dexterity, 3 × /wk, 1–1.5 h/ × , 26 wks, supervised, group	UPDRS-III: BW: + ; CON: 0; BW >> CON
Szefler-Derela et al. (2020) [69]	RCT	*N*: 40 Age: NA yrs HY: II–III MMSE: ≥ 24	UPDRS-III	Nordic walking, 2 × /wk, 1.5 h/ × , 6 wks, supervised, group	Standard rehabilitation, 2 × /wk, 0.75 h/ × , 6 wks, supervised, personal	UPDRS-III: NW: + ; CON: + ; NW >> CON
Franzoni et al. (2018) [70]**	RCT	*N*: 33 Age: > 50 yrs HY: I–IV MoCA: ≥ 26	NA	Nordic walking, 2 × /wk, 35–60 min/ × , 9 wks, supervised, group (6 familiarization sessions (35–50 min), 12 training sessions (1 h))	Free walking, 2 × /wk, 35–50 min/ × , 9 wks, supervised, group (6 familiarization sessions (35–50 min), 12 training sessions (1 h))	NA
Boxing (total *n* = 100)						
Sangarapillai et al. (2021) [71]	RCT	*N*: 40 Age: NA yrs HY: NA Cognition: NA	UPDRS-III	Boxing, 3 × /wk, 1 h/ × , 10 wks, supervised, group	PD Sensory Attention Focused Exercise, 3 × /wk, 1 h/ × , 10 wks, supervised, group	UPDRS-III: Boxing: − ; CON: + ; Boxing << CON
Domingos et al. (2022) [8]	RCT (pilot)	*N*: 29 Age: NA HY: NA MMSE: ≥ 24	Mini-BESTest, Feasibility	Boxing with kicks (BK), 1 × /wk, 1 h/ × , 10 wks, group, supervised	Boxing, 1 × /wk, 1 h/ × , 10 wks, group, supervised	Mini-BESTest: BK: + ; CON: + ; BK = CON Feasibility: The trainings were completed by 85% of the participants
Combs et al. (2013) [72]	RCT	*N*: 31 Age: ≥ 21 HY: NA Cognition: NA	NA	Boxing, 2–3 × /wk, 90 min/ × , 12 wks, group, supervised	Traditional exercise, 2–3 × /wk, 90 min/ × , 12 wks, group, supervised	NA
Climbing (total *n* = 48)						
Langer et al. (2021) [73]	RCT	*N*: 48 Age: NA yrs HY: 2–3 MMSE: ≥ 24	MDS-UPDRS-III	Top rope sport climbing (SC), 1 × /wk, 1.5 h/ × , 12 wks, supervised, group	Unsupervised physical training group, 150 min per week moderate exercise or 75 min per week vigorous exercise, resistance training 2 × /wk, balance exercises 3 × /wk, 12 wks	MDS-UPDRS-III: SC >> CON MDS-UPDRS-III (bradykinesia): SC >> CON MDS-UPDRS-III (rigidity): SC >> CON MDS-UPDRS-III (tremor): SC >> CON
Kayaking (total *n* = 48)						
Shujaat et al. (2014) [74]	RCT	*N*: 48 Age: 35–65 yrs HY: I–III Cognition: NA	NA	Kayaking, 6 × /wk, 75 min/ × , 4 wks, group, supervised	Strengthening exercise, 6 × /wk, 75 min/ × , 4 wks, group, supervised	NA

+ denotes a positive within-group effect, − denotes a negative within-group effect, 0 denotes no within-group effect, >> denotes a between-group effect favoring the group on the left of the sign, << denotes a between-group effect favoring the group on the right of the sign, = denotes a similar between-group effect in both groups

*ABC*, Activities-specific Balance Confidence Scale; *ACE*, Addenbrooke’s Cognitive Examination; *ACS*, Activities Constraint Scale; *ACS2*, Activity Card Sort; *AMTS*, Abbreviated Mental Test Score; *API*, anteroposterior index; *BBS*, Berg Balance Scale; *BDW-85-II*, muscle stability; *BK*, boxing with kicks; *BW*, brisk walking; *CON*, control group; *COP*, center of pressure; *DT*, dance therapy; *FAB*, frontal assessment battery; *FCS*, Falls Control Scale; *FMS*, Falls Management Scale; *HADS*, Hamilton Anxiety and Depression Scale; *HR*, heart rate; *HY*, Hoehn and Yahr stage; *MDS-UPDRS*, Movement Disorders Society Unified Parkinson Disease Rating Scale; *MoCA*, Montreal Cognitive Assessment; *MMSE*, Mini-State Examination; *N*, sample size; *NA*, not applicable; *OBI*, overall balance index; *PD*, Parkinson’s disease; *PDQ-8*, Parkinson’s Disease Questionnaire 8; *PDQ-39*, Parkinson’s Disease Questionnaire 39; *PDSS-2*, Parkinson’s Disease Sleep Scale-2; *PFS-16,* Parkinson’s Disease Fatigue Scale; *RCT*, randomized controlled trial; *SC*, sport climbing; *SIS*, Six-Item Screener; *S1DisAP*, magnitude of the posterior COP displacement; *S1DisML*, magnitude of the lateral COP displacement; *S1VelAP*, mean COP velocity in posterior direction; *S1VelML*, mean COP velocity in lateral direction; *TUG*, Timed Up and Go; *VAS*, Visual Analog Scale; *WQ*, wuqinxi qigong

## Results

**Fig. 1:**
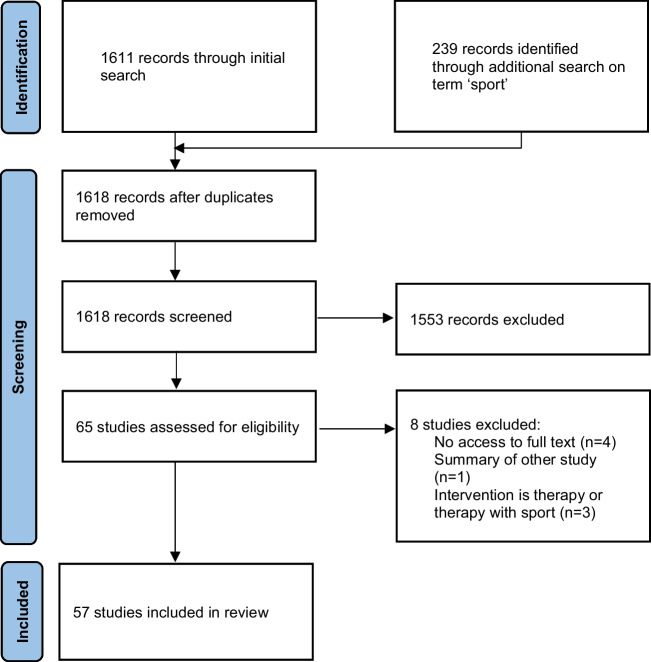
Flowchart of studies

Our search yielded 1618 unique publications. After title and abstract screening, 65 studies remained of which 57 were included in the final review (Fig. 1). These 57 studies described 53 exercise interventions (five studies described a secondary analysis, one study described two interventions) in a total of 2416 participants. The interventions varied in frequency (1–6 sessions/week), duration (mostly for 12 weeks or shorter with only a few that lasted longer (up to 2 years) [47]), and supervision (mostly supervised and in a group setting). Only few studies reported a single primary outcome measure. Overall, outcome measures were very heterogeneous but focused mostly on PD motor symptoms and balance.

Our results are summarized in Table 1. We will now briefly describe the main results of each exercise category separately.

### Yoga

Eight studies investigated the effect of (different types of) yoga, of which three reported a secondary analysis [24, 27, 75]. Yoga may alleviate non-motor problems such as anxiety and depressive symptoms, as was shown by the largest of the yoga studies including 138 participants [23, 27]. Other studies found a potential positive effect on motor problems such as balance [22, 26]. Conflicting evidence was found for the effects on quality of life [26, 27].

### Wuqinxi and Qigong

Ten studies investigated the effect of wuqinxi or qigong in a total of 477 people with PD. Most of the studies were in the pilot phase, mainly studying safety and feasibility. While wuqinxi and qigong were found to be safe and feasible for people with PD, we cannot draw firm conclusions on its effects. Most studies show preliminary effects (mostly within group differences) on physical functioning. The largest and longest study (*n* = 100, 6 months) compared Baduanjin qigong with an active control intervention (i.e., walking exercises, 7 times per week) and found no group differences [32]. However, motor symptoms, sleep, endurance, and balance improved in both groups. Interestingly, another study found that qigong was associated with a promising improvement of motor symptoms, sleep quality, balance, and endurance; these findings warrant further study [34].

### Tai Chi and Ai Chi

Ten studies investigated the effect of tai chi or ai chi; nine were original studies, while one reported a secondary analysis [76] and one study described two interventions [37]. All studies were supervised, except the study of Khuzema et al. [44] who provided the first session under supervision, while the remainder of the intervention was unsupervised. Tai chi and ai chi seem to improve balance (as measured with the Berg Balance Scale), as confirmed by three studies showing a between-group effect compared to a control group [39–41] and one study showing a within-group effect [44]. Another study showed a within-group improvement in balance only in the control group that received multimodal exercise training, but not in the tai chi group [45]. Also, some beneficial effects on motor symptoms (MDS-UPDRS) and health-related quality of life (PDQ-8) were found (Table 1).

### Dance

Sixteen studies investigated the effect of very different types of dance on PD ranging from tango, samba and folk, to ballroom dance. All interventions were supervised and delivered in a group setting, except for three studies, for which the group setting was unclear [35, 49, 52]. One study combined supervised group dance sessions with a dance program at home, which 71.5% of the participants complied with [50]. In another study, participants danced with their own partner [46]. Dance had a beneficial effect on (parts of the) MDS-UPDRS compared to the control group in four studies [47, 52, 56, 57, 60]. However, one study did not find such an effect [48]. The study of Duncan et al. [47] evaluated a 24-month intervention which showed that improvements were maintained over a prolonged period of time. However, this study was performed in a small group of only 10 participants. Finally, some studies emphasized that dance may improve not only physical functioning, but also cognitive and mental functioning. Results on other outcome measures were scarce.

### Pilates

Three studies investigated the effect of Pilates in 91 persons with PD. None of the studies specified a primary outcome and all secondary outcomes assessed motor function (i.e., UPDRS, TUG, BBS; Table 1). At the moment, there is insufficient evidence for Pilates.

### (Nordic) Walking

Six studies (one secondary analysis) investigated the effect of Nordic walking, while one study evaluated brisk walking. Most studies assessed the effects on PD motor symptoms, endurance, balance, and mobility and found beneficial effects favoring the (Nordic) walking group. Granziera et al. [67] compared Nordic walking with regular walking and found no added beneficial effect of Nordic walking. In contrast, three studies [65, 66, 70] also compared Nordic walking to a regular walking control group and did find an additional beneficial effect on gait and balance. Altogether, (Nordic) walking seems to improve functional mobility and gait. The effects on non-motor problems and quality of life remain unclear.

### Boxing

Three studies investigated the effect of (kick)-boxing. All of them included an active control group (PD Sensory Attention Focused Exercise, boxing without kicks and traditional exercise). Overall, boxing did not show many additional effects over exercise performed in the control groups. However, boxing seemed feasible and safe and within-group improvements on gait, balance, and motor symptoms were found. Given the limited number of studies, small sample sizes, and small contrast between the intervention and control groups, we cannot draw firm conclusions about the effects of boxing for people with PD.

### Rope Sport

Langer et al. [73] investigated the effect of sport climbing compared (12 weeks) to a control group that received unsupervised physical training in 48 people with PD. Climbing had a significant beneficial effect on motor symptoms (MDS-UPDRS-III) compared to the control group. Safety would seem to be an issue but no adverse events were reported and adherence to the climbing sessions was 99%. This novel intervention certainly deserves further study.

### Kayaking

Shujaat et al. [74] investigated the effect of kayaking six times per week for 4 weeks compared to a strengthening exercise group that exercised at the same frequency. Both interventions resulted in improvements in mobility.

## Discussion

Many different types of community-based exercise for people with PD are emerging and they are increasingly being studied. We here reviewed the evidence that is currently available for different types of community-based exercise in people with PD. This resulted in a heterogeneous overview of many types of exercise, study designs, and outcome measures. Despite limitations in study designs resulting in potential bias (e.g., selection bias), lack of generalizability, and modest effects sizes, all types of community-based exercise that we identified seem to be safe and feasible. We have to be more careful about the possible beneficial effects in light of the various limitations in study design, but overall, most programs appeared to have some beneficial effects in people with PD. But the positive experience with feasibility and adherence is perhaps the most important finding, because in order to achieve enduring benefits in a chronic and progressive disorder like PD, long-term adherence to exercise is critical, yet precisely this is challenging for many persons with PD, for a variety of reasons [9, 77]. Being able to choose from a range of activities to find an optimal fit between capability and preference will make exercise more enjoyable and thereby help to increase long-term adherence. Another interesting finding is that some types of community-based exercise (e.g., qigong, dance) are not only associated with the general physical benefits of exercise, but also seem to improve a range of non-motor symptoms and functioning, such as sleep, cognition, or emotional wellbeing. Because these symptoms of the disease generally respond less well to medical treatment [78, 79], this may have relevant effects on the quality of life of people with PD. Given the limitations in study design, it is uncertain whether these beneficial effects were driven by the intrinsic action of, e.g., qigong or dance, or whether these can also be ascribed — at least in part — to aspecific elements such as the social component of the group-based intervention.

An important question that remains to be answered is who should supervise community-based exercise for people with PD? In general, exercise instructors working in the community do not have PD-specific expertise, but physiotherapists (who are more skilled in PD care) are not qualified as exercise instructors of specific types of exercise like tai chi or dance. The main concerns here include safety and being able to adjust the activity to the capacities of the participant with PD. For physiotherapy, treatment by a therapist who is specialized in PD (through education and hands-on experience) has been shown to be more (cost-)effective than usual care physiotherapy [80, 81]. These benefits of specialized physiotherapy could be ascribed in part to the prevention of PD-related complications, including falls and fall-related injuries. We could argue that exercise instructors working with groups of people with PD should also receive some elementary education about PD, its progression, and safety. We mentioned the risk of falls as one potential but realistic concern, but another one is cardiovascular complications — persons with PD have a higher risk of cardiovascular disease [82], so strenuous exercise programs could potentially be associated with, e.g., myocardial infarction. This is not to say that all persons with PD should be screened for such cardiovascular risks prior to engaging in community-based exercise programs, but it is a factor that should be considered on a personal basis, based on a good understanding of PD and health issues in general. On the other hand, community-based exercise could rightly be considered as a leisure activity that is not part of regular healthcare. Should problems or symptoms emerge that make it difficult to participate in community-based exercise (or that need treatment), then a specialized physiotherapist could be consulted to receive further detailed personalized advice. Another option would be that a physiotherapist is consulted for a comprehensive evaluation and individually tailored exercise prescription, which is then subsequently performed in the community.

While this review is positive regarding the feasibility and potential effects of community-based exercise, it is also evident that the quality of the studies needs much improvement. For example, only a few studies specified a primary outcome. Many studies included multiple primary outcomes and some studies did not even differentiate between primary and secondary outcomes. In addition, the outcomes varied considerably across different studies. Most studies assessed physical functioning and motor symptoms, but non-motor symptoms and quality of life were often not included. It is essential to select outcome measures that are clinically relevant and meaningful for people with PD. This issue of relevance can sometimes be questioned, for example when joint angles or gait kinematics are the only outcomes (notwithstanding their merits for understanding the basic working mechanism of a given intervention). Consequently, the great variability in outcomes and the lack of primary, hypothesis-driven, outcomes make it difficult to draw any firm conclusions. Moreover, hardly any adequately powered studies were performed, and the large majority of studies used small sample sizes and short-duration interventions, further decreasing the strength of the evidence. Finally, the participants included in these studies were mainly in Hoehn and Yahr stages 1–3. Nine studies also allowed participants in Hoehn and Yahr stage 4. However, only very few people in Hoehn and Yahr stage 4 actually participated. Therefore, we cannot generalize these results to more advanced disease stages.

These challenges are not new and are partly inherent to studying non-pharmacological interventions which are inherently complex in nature. Challenges mainly relate to selecting appropriate interventions (both experimental and control), intervention dosing, adherence, and potential confounding. Other than in medication studies, dosing (including intensity, frequency, and duration) of a non-pharmacological intervention is very difficult and depends on many contextual factors (i.e., motivation and time of participant, expertise of the supervisor, etc.). It is also notoriously hard to measure adherence and the actual dosage received, often relying on self-report. Choosing an appropriate control intervention may be even more difficult, allowing for enough contrast while limiting nocebo effects (the negative effect of knowing to not be in the active intervention group). In the field of non-pharmacological interventions, there is no such thing as a true placebo intervention, and blinding of participants is hardly possible. Moreover, contamination between the treatment and control intervention and selection bias of participants interested in the subject occur frequently. While these challenges make these types of studies more complex, many of these can be overcome by carefully designing studies based on solid hypotheses about working mechanisms and based on pilot work that is abundantly available, as was shown by our present review. In general, we plea that the same quality criteria, as applied to pharmacological studies, should be applied to non-pharmacological interventions as well. The time is ripe for adequately designed (phase III) RCTs in the field of exercise in PD that not only assess efficacy but also to unravel the underlying working mechanisms.

In this review, we offered an extensive overview of the many studies that have been performed on community-based exercise in PD so far. We started this review from an initiative that intended to systematically search the literature for physiotherapy interventions and we adopted a pragmatic search strategy along the way. Therefore, we might have missed several trials. However, given the broad search strategy and systematic selection of articles, we believe that we covered the subject comprehensively. We would have liked to perform a meta-analysis on the results, based on which we could have drawn more rigorous conclusions about effect sizes and the comparative effectiveness of the different types of community-based exercise. We ultimately chose not to do this because of the large variety in interventions (types, dosing, duration), the great variety in outcome measures, and the overall small sample sizes, which would have reduced the power of such an analysis. Future studies are warranted to further study the clinical effects of community-based exercise, mainly in the long term, where we feel that the greatest promise lies. On the other hand, we doubt whether it is useful and needed to study every type of community-based exercise on (long-term) effectiveness, especially in a time of limited financial recourse for research. The effects of exercise have been widely studied in PD [2], and community-based exercise could be seen as way to keep people engaged in exercise in the long term [8]. This review shows that all types of community-based exercise seem to have some clinical effects as studied in many small pilot studies. At this point, we need innovative and efficient designs to push the field forward, beyond the pilot testing phase. The multiple arm multiple stage (MAMS) platform trials may offer an attractive solution by allowing efficient clinical evaluations across multiple interventions with one shared control group [83, 84]. An additional advantage of a MAMS trial is that a standard set of outcome measures allows for comparison between interventions and working mechanisms and that interim analyses make adaptations during the trial possible. In the meantime, different types of community-based exercise can be safely performed by people with PD, supporting them in managing their disease and maintaining or even improving quality of life.

## Conclusion

Many types of community-based exercise are available for people with PD, increasing access to exercise for more people. While feasibility has been largely shown, the evidence on the effects remains scarce for most of the interventions because of methodological constraints. However, the knowledge provided in this review may help people with PD to select the type and setting of exercise activity that matches best with their personal abilities and preferences. As such, these insights will contribute to an improved self-management of PD.

### Supplementary Information

Below is the link to the electronic supplementary material.

Supplementary file1 (DOCX 103 KB)
