# Supplementary material for: Exercise Made Accessible: the Merits of Community-Based Programs for Persons with Parkinson’s Disease
Source: Curr Neurol Neurosci Rep. 2023 Oct 4;23(11):695–715. doi: 10.1007/s11910-023-01303-0 (PMC10673991; doi:10.1007/s11910-023-01303-0)
Supplement: Supplementary file 1 — Supplementary file1 (DOCX 103 KB) [file 11910_2023_1303_MOESM1_ESM.docx]

| **Authors (year)** | **Secondary outcomes** | **Result secondary outcome** | |
| --- | --- | --- | --- |
| **Yoga** | | | |
| Ni (2016)**(1) | MDS-UPDRS, 1RM, PDQ-39 | Bradykinesia (MDS-UPDRS): Yoga >> CON; Muscle strength (1RM biceps, chest, leg, hip, calf): Yoga >> CON; Power (peak power 1RM leg): Yoga >> CON; Power (peak power 1RM biceps, chest, hip, calf): 0; QoL (PDQ-39): Yoga >> CON; | |
| Kwok (2019)(2) | MDS-UPDRS III, TUG, HWS, PDQ-8 | MDS-UPDRS III: Yoga >> CON TUG: 0 HWS: Yoga >> CON PDQ-8: Yoga >> CON | |
| Van Puymbroeck (2018)(3) | MDS-UPDRS, HY, Mini-BESTest, FGA, FOG-Q | MDS-UPDRS: Yoga +; CON 0 HY: Mini-BESTest: Yoga +; CON + FGA: Yoga >> CON FOG-Q: Yoga +; CON 0 | |
| Cherup (2021)(4) | Balance error scoring system, dynamic posturography, TUG, FES-I | Balance error scoring system: 0 Dynamic posturography: Yoga +; CON + TUG: 0 FES-I: 0 | |
| Walter (2019)**(5) | - | - | |
| Kwok (2022)**(6) | MDS-UPDRS I, MDS-UPDRS II, MDS-UPDRS III, HADS, PDQ-8 | MDS-UPDRS I: Yoga >> CON (after intervention (T1) and three months after intervention (T2)), Yoga: + (T1 & T2) MDS-UPDRS II: Yoga >> CON (T1), Yoga: + (T1 & T2), CON: + (T2) HADS-anxiety: Yoga >> CON (T1 & T2), Yoga: + (T1 & T2), CON: + (T2) HADS-depression: Yoga >> CON (T1 & T2), Yoga: + (T1 & T2) PDQ-8: Yoga >> CON (T1 & T2), Yoga: + (T1 & T2) | |
| Elangovan (2020)(7) | MDS-UPDRS III, Postural stability, Gait kinematics, Flexibility of trunk and lower limbs | MDS-UPDRS III: Yoga = CON Postural stability: Yoga > CON Gait kinematics: Yoga = CON Flexibility: Yoga = CON | |
| Ni (2016)(8) | BBS, Mini-BESTest, SLS, Postural Sway, TUG, functional reach, 10MWT, Leg press - strength, leg press - peak power | BBS: PWT: +; Yoga: +; PWT > CON; Yoga > CON Mini-BESTest: PWT: +; Yoga: +; PWT > CON; Yoga > CON TUG: PWT: +; Yoga: +; PWT > CON; Yoga = CON Functional reach (more affected): PWT: +; Yoga: 0; PWT > CON; Yoga = CON Functional reach (less affected): PWT: +; Yoga: +; PWT >CON; Yoga > CON SLS (more affected): PWT: +; Yoga: 0; PWT = CON; Yoga = CON SLS (less affected): PWT: 0; Yoga: +; PWT = CON; Yoga = CON 10MWT (Uwalk speed): PWT: +; Yoga: +; CON: 0; PWT > CON; Yoga > CON 10MWT (Mwalk speed): PWT: +; Yoga: +; CON: 0; PWT > CON; Yoga > CON Leg press (strength): PWT: +; Yoga: +; CON: 0; PWT > CON; Yoga >CON Leg press (peak power): PWT: +; Yoga: +; CON: 0; PWT > CON; Yoga > CON Postural sway: Yoga: + | |
| **Wuqinxi and Qigong** | | | |
| Wang (2020)(9) | Purdue pegboard test, soda pop test, PDQ-39 | PPT: Wuqinxi >> CON Soda pop test: Wuqinxi +; CON + PDQ-39: Wuqinxi +; CON + | |
| Shen (2021)(10) | UPDRS III / TUG | MDS-UPDRS III: Wuqinxi: +; stretching: 0 TUG: Wuqinxi: +; stretching: - | |
| Wan (2021)(11) | Reaction time test / TUG / one legged blind balance test / 10MWT (normal and fast speed) / Sit and reach / RoM schoulder, knee, hip | Reaction time test: Qigong: 0; CON: - One-legged blind balance test left: Qigong: +; CON: 0 One-legged blind balance test right: Qigong: +; CON: 0 TUG: Qigong: +; CON: 0; Qigong >> CON Normal speed 10MWT (stride length): Qigong: +; CON: 0 Normal speed 10MWT (cadence): Qigong: 0; CON: 0 Normal speed 10MWT (gait velocity): Qigong: +; CON: 0; Qigong >> CON Fast speed 10MWT (stride length): Qigong: 0; CON: 0 Fast speed 10MWT (cadence): Qigong: 0; CON: 0 Fast speed 10MWT (gait velocity): Qigong: +; CON: 0; Qigong >> CON Sit and reach: Qigong: +, CON: 0 Knee flexion left: Qigong: +; CON: 0; Qigong >> CON Knee flexion right: Qigong: +; CON:0; Qigong >> CON Hip flexion left: Qigong: 0; CON: -; Qigong >> CON Hip flexion right: Qigong: 0; CON: 0 Hip extension left: Qigong: +; CON: 0; Qigong >> CON Hip extension right: Qigong: +; CON: 0; Qigong >> CON Shoulder joint left: Qigong: +; CON: 0; Qigong >> CON Shoulder joint right: Qigong: +; CON: 0; Qigong >> CON | |
| Xiao (2016)(12) | NA | NA | |
| Liu (2016)(13) | 9-holed instrument test, TUG, turn-over-jar test, one-legged blind balance test | Turn-over-jar test (left): Qigong: +; CON: 0 Turn-over-jar test (right): Qigong: +; CON: 0 TUG: Qigong: +; CON: 0 9-holed instrument test (left): Qigong: 0; CON: 0 9-holed instrument test (right): Qigong: 0; CON: 0 One-legged blind balance test (left): Qigong: +; CON: 0 One-legged blind balance test (right): Qigong: +; CON: 0 | |
| Moon (2020)(14) | PDSS-2, Actigraph, PFS, GAI, GDS, MMSE, FAB, CDT, TMTA, TMTB, NMSQ, PDQ, MDS-UPDRS (I-III) | PDSS-2 (total): Qigong: +; CON: + PDSS-2 (motor symptoms): Qigong: +; CON: 0 PDSS-2 (PD symptoms): Qigong: 0; CON: 0 PDSS-2 (disturbed sleep): Qigong: 0; CON: + Actigraph (sleep efficiency): Qigong: 0; CON: 0 Actigraph (total time in bed): Qigong: 0; CON: 0 Actigraph (total sleep time): Qigong: 0; CON: 0 Actigraph (wake after sleep onset): Qigong: 0; CON: 0 Actigraph (awakenings): Qigong: 0; CON: 0 Actigraph (avg. awakening): Qigong: 0; CON: 0 PFS-16: Qigong: 0; CON: 0 GAI: Qigong: 0; CON: 0 GDS: Qigong: 0; CON: + MMSE: Qigong: 0; CON: 0 CDT: Qigong: 0; CON: 0 TMT-A: Qigong: 0; CON: 0 TMT-B: Qigong: 0; CON: 0 NMSQ: Qigong: +; CON: + PDQ-39: Qigong: 0; CON: + MDS-UPDRS (I): Qigong: 0; CON: 0 MDS-UPDRS (II): Qigong: 0; CON: 0 MDS-UPDRS (III): Qigong: 0; CON: 0 | |
| Li (2022)(15) | MDS-UPDRS III, MDSUPDRS, TUGT, MiniBESTest, PDQ-39 | MDS-UPDRS III: WQ: +; CON: 0; WQ = CON MDS-UPDRS: WQ: +; CON: 0; WQ = CON  TUG: WQ: +; CON: 0; WQ = CON Mini-BESTest: WQ: +; CON: 0; WQ = CON PDQ-39: WQ: +; CON: 0; WQ = CON | |
| Li (2022)(15) | Gait constant and high speed (stride length, stride frequency, gait velocity; ) Lower-limb joint range of motion (hip flexion/extension, knee flexion), Timed Up and Go (TUG), Motor function assessment of the Unified Parkinson's Comprehensive Rating Scale (UPCRS) | Constant speed stride length: Qigong: +; CON: 0 Constant speed stride frequency: Qigong: 0; CON: 0 Constant gait velocity: Qigong: +; CON: 0 High speed stride length: Qigong: +; CON: 0 High speed stride frequency: Qigong: 0; CON: 0 High speed gait velocity: Qigong: +; CON: 0 Hip flexion left: Qigong: +; CON: 0 Hip flexion right: Qigong: +; CON: 0 Hip extension left: Qigong: +; CON: 0 Hip extension right: Qigong: +; CON: 0 Knee flexion left: Qigong: +; CON: 0 Knee flexion right: Qigong: +; CON: 0 TUG: Qigong: +; CON: 0 Motor function assessment UPCRS: Qigong: +; CON: 0 | |
| Wang (2022)(16) | HADS, PDSS, PDQ-39, UPDRS-III, IPAQ, Timed Up-and-Go Test (TUG), MoCA and inhibition capacity (motor inhibition tasks). | HADS: Wuqinxi: 0; CON: 0 PDSS-2: Wuqinxi: 0; CON: 0 PDQ-39: Wuqinxi: 0; CON: 0 MDS-UPDRS-III: Wuqinxi: 0; CON: 0 IPAQ: Wuqinxi: +; CON: 0; Wuqinxi >> CON TUG: Wuqinxi: 0; CON: 0 MoCA: Wuqinxi: 0; CON: 0 Stop signal reaction time (ms): Wuqinxi: +; CON: 0;  Context effects (ms): Wuqinxi: 0; CON: 0 | |
| Amano (2013)*(17) | Gait cycle: (1) cadence, (2) gait velocity, (3) step length, (4) step duration, (5) swing time, (6) double limb support time, (7) gait asymmetry UPDRS-III | Gait (cadence): Qigong = CON Gait (velocity): Qigong = CON Gait (step length): Qigong = CON Gait (step duration): Qigong = CON Gait (swing time): Qigong = CON Gait (double limb support time): Qigong = CON Gait (gait asymmetry): Qigong = CON MDS-UPDRS-III: Qigong = CON | |
|  | | | **Tai Chi and Ai Chi** |
| Amano (2013)*(17) | Gait cycle: (1) cadence, (2) gait velocity, (3) step length, (4) step duration, (5) swing time, (6) double limb support time, (7) gait asymmetry UPDRS-III | Gait (cadence): Tai Chi = CON Gait (velocity): Tai Chi = CON Gait (step length): Tai Chi = CON Gait (step duration): Tai Chi = CON Gait (swing time): Tai Chi = CON Gait (double limb support time): Tai Chi = CON Gait (gait asymmetry): Tai Chi = CON MDS-UPDRS-III: Tai Chi = CON | |
| Amano (2013)*(17) | Gait cycle: (1) cadence, (2) gait velocity, (3) step length, (4) step duration, (5) swing time, (6) double limb support time, (7) gait asymmetry UPDRS-III | Gait (cadence): Tai Chi = CON Gait (velocity): Tai Chi = CON Gait (step length): Tai Chi = CON Gait (step duration): Tai Chi = CON Gait (swing time): Tai Chi = CON Gait (double limb support time): Tai Chi = CON Gait (gait asymmetry): Tai Chi = CON MDS-UPDRS-III: Tai Chi = CON | |
| Li (2014)(18) | PDQ - 8, Vitality Plus Scale (Summary score), UPDRS-III, 50-foot speed walk test, 3-month postintervention follow-up (improved, not improved) | PDQ-8: Tai-chi >> Resistance, Tai-chi >> CON VPS: Tai-chi = Resistance, Tai-chi >> CON | |
| Gao (2014)(19) | Falls | Falls: Tai Chi >> CON Average times of falls: Tai Chi >> CON | |
| Kurt (2018)(20) | NA | NA | |
| Pérez de la Cruz (2017)(21) | BBS, Tinetti Scale (TS), Five Times Sit-to-Stand Test (FTSTS), TUG and the UPDRS | BBS: Ai Chi: +; CON: 0; Ai Chi >> CON Tinetti Scale: Ai Chi: +; CON: 0; Ai Chi >> CON FTSTS: Ai Chi: 0; CON: 0 TUG: Ai Chi: +; CON: 0; Ai Chi >> CON MDS-UPDRS (1-3): Ai Chi: +; CON: 0; Ai Chi >> CON MDS-UPDRS (4): Ai Chi: 0; CON: 0 MDS-UPDRS (total): Ai Chi: +; CON: 0; Ai Chi >> CON | |
| Pérez de la Cruz (2018)(22) | VAS, TUG, Five Times Sit-to-Stand Test (FSTST), single leg standing, Yesavage Test, PDQ-39 | VAS: Ai Chi +; CON: 0; Ai Chi >> CON TUG: Ai Chi +; CON: 0; Ai Chi >> CON FTSTS: Ai Chi +; CON: 0; Ai Chi >> CON Single leg standing (left): Ai Chi: +; CON: 0; Ai Chi >> CON Single leg standing (right): Ai Chi: +; CON:0; Ai Chi >> CON Yesavage: Ai Chi: +; CON: 0; Ai Chi >> CON PDQ-39 (mobility): Ai Chi = CON PDQ-39 (emotional well-being): Ai Chi = CON PDQ-39 (cognitive impairment): Ai Chi = CON PDQ-39 (bodily discomfort): Ai Chi = CON PDQ-39 (ADL): Ai Chi = CON PDQ-39 (stigmas): Ai Chi = CON PDQ-39 (social support): Ai Chi >> CON PDQ-39 (communication) Ai Chi = CON | |
| Pérez de la Cruz (2019)**(23) | SF-36, Geriatric Depression Scale (GDS), VAS | VAS: Ai Chi +; CON: +; Ai Chi >> CON GDS: Ai Chi >> CON SF-36: Ai Chi +; CON: 0; Ai Chi >> CON | |
| Khuzema (2020)*(24) | BBS, 10MWT, TUG | BBS: Tai Chi: +; Yoga: +; CON: +; Tai Chi = Yoga = CON 10MWT: Tai Chi: +; Yoga: +; CON: +; Tai Chi = Yoga = CON TUG: Tai Chi: +; Yoga: +; CON: +; Tai Chi = Yoga = CON | |
| Zhang (2015)(25) | MDS-UPDRS III, stride length, gait velocity, TUG | MDS-UPDRS-III: Tai chi: +; CON: +; Tai chi = CON stride length: Tai chi: +; CON: +; Tai chi = CON gait velocity: Tai chi: +; CON: +; Tai chi = CON TUG: Tai chi: +; CON: +; Tai chi = CON | |
| Poier (2019)*(26) | Brief Multidimensional Life Satisfaction Scale (BMLSS), Inner Correspondence and feelings of Peaceful Relief (ICPH), Perceived impairment in everyday life was measured with a numeric rating scale (0-100), Expected effectiveness of the intervention was measured with a numeric rating scale (0-100) | BMLSS: Tai chi: 0; CON: 0; Tai chi = CON ICPH: Tai chi: 0; CON: 0; Tai chi = CON Perceived impairment in everyday life: Tai chi: 0; CON: 0; Tai chi = CON Expected effectiveness of the intervention: Tai chi: 0; CON: 0; Tai chi = CON | |
|  | | | **Dance** |
| Duncan (2014)(27) | Mini-BESTest, gait velocity (forward and backward), TUG, 6MWT, MDS-UPDRS II, MDS-UPDRS I, and Freezing of Gait Questionnaire | Mini-BESTest: AT >> CON MDS-UPDRS II: AT >> CON MDS-UPDRS I: AT >> CON Forward walking velocity: AT = CON Backward walking velocity: AT = CON TUG: AT = CON dTUG: AT = CON; AT: +; CON: - 6MWT: AT = CON; CON: - FOG-Q: AT = CON | |
| Rios Romenets (2015)(28) | MDS-UPDRS-4, Mini-BESTest Dual task TUG, TUG, Falls questionnaire Freezing of Gait Questionnaire (FOG Q). Purdue Pegboard for assessment of upper extremity function, MoCA, BDI, Apathy Scale (AS), Krupp Fatigue Severity Scale. PDQ-39, Clinical Global Impression of Change (CGI-C), exit questionnaire ranking level of enjoyment and over- all satisfaction with their dance/exercise program, scored from 1 (strongly agree) to 5 (strongly disagree), with open questions about willingness continuing practicing tango | MDS-UPDRS IV: Tango = CON Mini-BESTest: Tango > CON dTUG: Tango: +; Tango > CON TUG: Tango: +; Tango > CON Falls: Tango = CON FOG-Q: Tango = CON PPT: Tango = CON MoCA: Tango = CON BDI: Tango = CON AS: Tango = CON Krupp: Tango > CON PDQ-39: Tango = CON CGI-c patient: Tango = CON CGI-c examiner: Tango > CON | |
| Hashimoto (2015)(29) | TUG, BBS, FAB, MRT, Apathy Scale, Self-Rating Depression Scale, UPDRS, heart rate | TUG (time): Dance: +; PD exercise: +; CON: + TUG (step nr): Dance: +; PD exercise: +; CON: 0 BBS: Dance: +; PD exercise: 0; CON: 0 FAB: Dance: +; PD exercise: +; CON: 0 MRT (response time): Dance: +; PD exercise: +; CON: 0 MRT (number of correct answers): Dance: 0; PD exercise: 0; CON: 0 Apathy Scale: Dance: +; PD exercise: 0; CON: 0 SDS: Dance: +; PD exercise: 0; CON: 0 MDS-UPDRS: Dance: +; PD exercise: 0; CON: - | |
| Shanahan (2017)(30) | MDS-UPDRS, PDQ-39, 6MWT, miniBESTest | No significant difference between groups at baseline or postintervention. | |
| Hulbert (2017)(31) | 3D motion analysis: - latency - rotation - weight transfer - clinical | Latency head was longer in CON Greater rotation of pelvis in CON Slower movement of first and second foot in CON Minimal change in Dance for these outcomes Centre of Mass, Turn Time, Clinical Measures: Dance = CON | |
| Lee (2018)(32) | PDQL, BBS, BDI | PDQL (Parkinson's symptoms): Turo = CON PDQL (Systemic symptoms): Turo >> CON PDQL (Social functioning): Turo >> CON PDQL (Emotional functioning): Turo = CON PDQL (total): Turo >> CON BDI: Turo = CON BBS: Turo = CON | |
| Michels (2018)(33) | H&Y, MDS-UPDRS, BBS, TUG, MOCA, PDQ-39, BDI, FSS, VAFS | The study was not powered to assess whether any of the observed differences were statistically significant. | |
| Rawson (2019)(34) | FWD velocity, BWD velocity, 6MWT, Mini-BESTest, MDS-UPDRS III, PDQ-39 | FWD velocity: Tango >> CON; Treadmill: + BWD velocity: Tango = Treadmill = CON; Tango: +; Treadmill: +; CON: + 6MWT: Tango = Treadmill = CON; Tango: +; Treadmill: + Mini-BESTest: Treadmill: +; CON: - MDS-UPDRS III: CON: + PDQ-39: Stretching: +; Tango & Treadmill << CON | |
| Kalyani (2019)(35) | Addenbrooke's Cognitive Examination (ACE), MDS-UPDRS, NIH Toolbox, TMT, HADS, PDQ-39 | ACE: unclear NIH Toolbox (executive function - flanker inhibitory control & attention test): Dance = CON NIH Toolbox (executive function - dimensional change card sort test): Dance = CON NIH Toolbox (episodic memory - picture sequence memory test): Dance = CON NIH Toolbox (episodic memory - auditory verbal learning test): Dance >> CON NIH Toolbox (language - picture vocabulary test): Dance = CON NIH Toolbox (processing speed - pattern comparison processing speed test): Dance = CON TMT-A: Dance = CON TMT-B: Dance >> CON TMT (B-A): Dance >> CON HADS (depression): Dance >> CON HADS (anxiety): Dance >> CON MDS-UPDRS I: Dance >> CON PDQ-39 (mobility): Dance = CON PDQ-39 (activities of daily living): Dance >> CON PDQ-39 (emotional well-being): Dance >> CON PDQ-39 (stigma): Dance = CON PDQ-39 (social support): Dance = CON PDQ-39 (cognition): Dance >> CON PDQ-39 (communication): Dance = CON PDQ-39 (bodily discomfort): Dance = CON PDQ-39 (summary index): Dance >> CON MDS-UPDRS II: Dance >> CON | |
| Tillmann (2019)(36) | HY, UPDRS, BBS, PDQ-39 | MDS-UPDRS total: CON: -; Dance: +; Dance >> CON Mental estate: CON: 0; Dance: 0; Dance >> CON Daily activities: CON: -; Dance: +; Dance >> CON Motor examination: CON: 0; Dance: +; Dance >> CON Complications in therapy: CON: -; Dance: 0; Dance = CON BBS: CON: 0; Dance: +; Dance >> CON PDQ-39 (mobility): CON: 0; Dance: +; Dance = CON PDQ-39 (daily activity): CON: 0; Dance: 0; Dance = CON PDQ-39 (emotional): CON: 0; Dance: 0; Dance = CON PDQ-39 (stigma): CON: 0; Dance: 0; Dance = CON PDQ-39 (support): CON: 0; Dance: 0; Dance = CON PDQ-39 (cognition): CON: 0; Dance: 0; Dance = CON PDQ-39 (communication): CON: 0; Dance: 0; Dance = CON PDQ-39 (discomfort): CON: 0; Dance: 0; Dance = CON PDQ-39 (total): CON: 0; Dance: 0; Dance = CON | |
| Frisaldi (2021)(37) | 6MWT, TUG, Mini-BESTest, NFOG-Q, MoCA, TUG-DT, PDQ-39-SI, BDI, STAI, FES-I, King's PD pain scale, PFS-16 | 6MWT: Dance = CON NFOG-Q: Dance = CON Mini-BESTest: Dance = CON TUG: Dance = CON MoCA: Dance = CON dTUG: Dance = CON BDI: Dance = CON FES-I: Dance = CON PDQ-39: Dance = CON STAI-Y1: Dance = CON STAI-Y2: Dance << CON PD pain scale: Dance = CON PFS-16: Dance = CON | |
| Foster (2013)(38) | NA |  | |
| Kunkel (2017)(39) | PDQ-39, SS180, TUG, ABC, 6MWT | PDQ-39 (3 & 6 months): Dance = CON SS180 (seconds, 3 & 6 months): Dance = CON SS180 (step count, 3 months): Dance = CON SS180 (step count, 6 months): Dance << CON TUG (3 months): Dance << CON TUG (6 months): Dance << CON ABC (3 & 6 months): Dance = CON 6MWT (3 months): Dance = CON | |
| Poier (2019)*(26) | Brief Multidimensional Life Satisfaction Scale (BMLSS), Inner Correspondence and feelings of Peaceful Relief (ICPH), Perceived impairment in everyday life was measured with a numeric rating scale (0-100), Expected effectiveness of the intervention was measured with a numeric rating scale (0-100) | BMLSS: Tango: 0; CON: 0; Tango = CON ICPH: Tango: 0; CON: 0; Tango = CON Perceived impairment in everyday life: Tango: 0; CON: 0; Tango = CON Expected effectiveness of the intervention: Tango: 0; CON: 0; Tango = CON | |
| Solla (2019)(40) | UPDRS-III, 6MWT, BBS, TUG, FTSST, BST, SRT, stride length, gait speed, cadence, number of straight walks, straight walking time, GFI, PFS-16, BDI-II, SAS, MOCA | MDS-UPDRS-III: Ballu Sardu: +; CON: 0; Ballu Sardu >> CON 6MWT: Ballu Sardu: +; CON: 0; Ballu Sardu >> CON BBS: Ballu Sardu: +; CON: 0; Ballu Sardu >> CON TUG: Ballu Sardu: +; CON: +; Ballu Sardu >> CON FTSTS: Ballu Sardu: +; CON: +; Ballu Sardu >> CON BST: Ballu Sardu: +; CON: 0; Ballu Sardu >> CON SRT: Ballu Sardu: 0; CON: 0; Ballu Sardu = CON stride length: Ballu Sardu: +; CON: 0; Ballu Sardu >> CON gait speed: Ballu Sardu: +; CON: 0; Ballu Sardu >> CON cadence: Ballu Sardu: +; CON: +; Ballu Sardu = CON number of straight walks: Ballu Sardu: +; CON: 0; Ballu Sardu >> CON straight walking time: Ballu Sardu: 0; CON: 0; Ballu Sardu = CON GFI: Ballu Sardu:0+; CON: -; Ballu Sardu >> CON PFS-16: Ballu Sardu: 0; CON: 0; Ballu Sardu = CON BDI-II: Ballu Sardu: +; CON: 0; Ballu Sardu >> CON SAS: Ballu Sardu:0+; CON: -; Ballu Sardu >> CON MoCA: Ballu Sardu: +; CON: 0; Ballu Sardu >> CON | |
| Li (2022)(41) | UPDRS, BBS, TUG, PPT | MDS-UPDRS: Yang-ge: +; CON(I): +; CON(II): +; Yang-ge = CON(I) = CON(II) BBS: Yang-ge: +; CON(I): +; CON(II): +; Yang-ge = CON(I) = CON(II) TUG: Yang-ge: +; CON(I): +; CON(II): + PPT: Yang-ge: +; CON(I): 0; CON(II): 0 | |
|  | | | **Pilates** |
| Maciel (2020)(42) | UPDRS-III, Schwab & England, POMA, TUG, Parkinson Activity Scale, Functional Reach Test | MDS-UPDRS-III: Pilates: +, CON: -, Pilates = CON Schwab & England: Pilates: 0; CON: 0; Pilates = CON POMA: Pilates: +; CON: 0; Pilates >> CON TUG: Pilates: +; CON: 0; Pilates >> CON PAS: Pilates: +; CON: 0; Pilates >> CON FRT: Pilates: +; CON: 0; Pilates >> CON | |
| Mollinedo-Cardalda (2018)(43) | 30 second chair stand, five sit ups, UPDRS-III, TUG | 30 Second Chair Stand: Pilates (intervention): +; Pilates >> CON (intervention); Pilates = CON (follow-up) Five Sit Ups: ; Pilates: + (intervention); Pilates >> CON (intervention); Pilates = CON (follow-up) MDS-UPDRS-III: Pilates = CON (intervention); Pilates = CON (follow-up) TUG (total): Pilates: + (intervention); Pilates >> CON (intervention); Pilates = CON (follow-up) | |
| Göz (2021)(44) | Limits of stability (LOS), Tandem walk test, Walk across test, Sit to stand test, Trunk Impairment Scale (TIS), BBS | TIS: Pilates: 0; Pilates + ET: 0; CON: 0 BBS: Pilates: 0; Pilates + ET: 0; CON: 0 LOS reaction time(sec): Pilates: +; Pilates + ET: +; CON: 0 LOS movement velocity (deg/sec): Pilates: 0; Pilates + ET: 0; CON: 0 LOS endpoint (%): Pilates: +; Pilates + ET: 0; CON: 0 LOS ESM (%): Pilates: 0; Pilates + ET: 0; CON: 0 LOS direction control (%): Pilates: 0; Pilates + ET: 0; CON: 0 Tandem walk test step width (cm): Pilates: 0; Pilates + ET: 0; CON: 0 Tandem walk test speed (cm/sec): Pilates: 0; Pilates + ET: 0; CON: 0 Tandem walk test end sway (deg/sec): Pilates: 0; Pilates + ET: +; CON: 0 Walk across test step step width (cm): Pilates: 0; Pilates + ET: 0; CON: 0 Walk across test step length (cm): Pilates: 0; Pilates + ET: 0; CON: 0 Walk across test speed (cm/sec): Pilates: +; Pilates + ET: +; CON: 0 Walk across test step symmetry (%): Pilates: 0; Pilates + ET: 0; CON: 0 Sit to stand test weight transfer (sec): Pilates: 0; Pilates + ET: 0; CON: 0 Sit to stand test velocity (deg/sec): Pilates: 0; Pilates + ET: 0; CON: 0 Sit to stand test rising index (%): Pilates: 0; Pilates + ET: +; CON: 0 Sit to stand test symmetry (%): Pilates: 0; Pilates + ET: 0; CON: 0 | |
|  | | | **(Nordic) walking** |
| Cugusi (2015)(45) | UPDRS-III, 6MWT, Five Times Sit to Stand, Hand-grip, BBS, TUG, Sit and reach, Back Scratch, PFS-16, BDI-II, Apathy Scale, Non Motor Symptoms Scale | MDS-UPDRS-III: NW: +; CON: -; NW >> CON 6MWT: NW: +; CON: 0; NW >> CON Five Time Sit to Stand: NW: +; CON: 0; NW >> CON Hand-grip: NW: 0; CON: 0; NW = CON BBS: NW: +; CON: -; NW >> CON TUG: NW: +; CON: -; NW >> CON Sit and reach: NW: +; CON: 0; NW >> CON Back scratch: NW: 0; CON: 0; NW = CON PFS-16: NW: +; CON: 0; NW >> CON BDI-II: NW: +; CON: 0; NW >> CON Apathy Scale: NW: +; CON: -; NW >> CON Non-motor symptoms scale: NW: +; CON: -; NW >> CON | |
| Monteiro (2017)(46) | UPDRS-III, TUG (self selected speed), TUG (forced speed), BBS, Self selected speed (SSW), Locomotor rehabilitation index (LRI) | TUG (self selected speed, baseline): NW >> CON TUG (forced speed, baseline): NW >> CON TUG (forced speed, pre-training, post familiarization): NW >> CON SSW (baseline): NW >> CON SSW (pre-training, post familiarization): NW >> CON SSW (post-training): NW >> CON LRI (baseline): NW >> CON LRI (pre-training, post familiarization): NW >> CON LRI (post-training): NW >> CON MDS-UPDRS-III: NW: +; CON: +; NW = CON BBS: NW: +; CON: +; NW = CON | |
| Bang (2017)(47) | UPDRS-III, BBS, TUG, 10MWT, 6MWT | MDS-UPDRS-III: NW: +; CON: +; NW >> CON BBS: NW: +; CON: +; NW >> CON TUG: NW: +; CON: +; NW >> CON 10MWT: NW: +; CON: +; NW >> CON 6MWT: NW: +; CON: 0; NW >> CON | |
| Granziera (2021)(48) | 6MWT, 10MWT, POMA T, TUG, PDQ-39, PFS-16, BDI, HAM-A, NMS | 6MWT: NW = CON 10MWT: NW = CON POMA T: NW = CON TUG: NW = CON PDQ-39: NW = CON PFS-16: NW = CON BDI: NW = CON NMS: NW = CON | |
| Mak (2021)(49) | Fast gait speed (FGS), TUG, 6MWT, Mini-BESTest | FGS: BW: +; CON 0; BW >> CON TUG: BW: +; CON: 0; BW >> CON 6MWT: BW: +; CON: 0; BW >> CON Mini-BESTest: BW: +; CON: 0; BW >> CON | |
| Szefler-Derela (2020)(50) | Dynamic Gait Index (DGI), TUG, PDQ-39 | DGI: NW: +; CON: +; NW = CON TUG: NW: +; CON: +; NW = CON PDQ-39: NW: +; CON: +; NW = CON | |
| Franzoni (2018)**(51) | COP parameters, BBS | BBS: NW: +; CON: +; NW = CON Postural balance; NW: + | |
|  | | | **Boxing** |
| Sangarapillai (2021)(52) | Stride length, stride velocity, CHAMPS, PDQ-39 | Stride length: BOX << CON Stride velocity: BOX << CON CHAMPS: BOX = CON PDQ-39: BOX >> CON | |
| Domingos (2022)(53) | FES-I, ABC, 6MWT, TUG, PDQ-39 | FES-I: BK: 0; CON: 0; BK = CON ABC: BK: 0; CON: 0; BK = CON 6MWT: BK: 0; CON: 0; BK = CON TUG: BK: 0; CON: -; BK = CON dTUG: BK: 0; CON: 0; BK = CON PDQ-39: BK: 0; CON: +; BK = CON | |
| Combs (2013)(54) | BBS, ABC, TUG, dTUG, gait velocity, 6MWT, PDQL | BBS: BOX: +; CON: +; BOX = CON ABC: BOX: 0; CON: +; BOX < CON TUG: BOX: +; CON: +; BOX = CON dTUG: BOX: +; CON: +; BOX = CON Gait velocity: BOX: +; CON: 0; BOX = CON 6MWT: BOX: +; CON: 0; BOX = CON PDQL: BOX: +; CON: +; BOX = CON | |
| **Climbing** | | | |
| Langer (2021)(55) | Feasibility | Feasibility: yes | |
| **Kayaking** | | | |
| Shujaat (2014)(56) | MPAS III A/B (bed mobility), rotation of cervical and thoracolumbar spine | MPAS III A: Kayak: +; CON: + MPAS III B: Kayak: +; CON: + Rotation cervical spine (left): Kayak: +; CON: + Rotation cervical spine (right): Kayak: +; CON: + Rotation thoracolumbar spine (left): Kayak: +; CON: + Rotation thoracolumbar spine (right): Kayak: +; CON: + | |

* Denotes a study that reported on multiple types of sports and was included for more than one sport in the table,** Denotes a study reporting a secondary analysis of a study that is described elsewhere in the Table, + Denotes a positive within-group effect, - Denotes a negative within-group effect, 0 Denotes no within-group effect, >> Denotes a between-group effect favouring the group on the left of the sign, << Denotes a between-group effect favouring the group on the right of the sign, = Denotes a similar between-group effect in both groups

10MWT= 10 meter walk test, 1RM= One-Repetition Maximum, 6MWT= 6 minute walk test, ABC= Activities-specific Balance Confidence Scale, ACE= Addenbrooke's Cognitive Examination, ADL= activities of daily living, AS= Apathy Scale, AT= Argentine Tango, avg.= average, BBS= Berg Balance Scale, BDI= Beck Depression Inventory, BK= BOX with kicks, BMLSS= Brief Multidimensional Life Satisfaction Scale, BOX= BOX, BST= Back Scratch Test, BW= brisk walking, BWD= backward, CDT= 10-point Clock Drawing Test, CGI-c= Clinical Global Impression of Change, CHAMPS= Community Health Activities Model Program for Seniors, cm= centimeter, CON= control group, deg= degrees, DGI= Dynamic Gait Index, dTUG= Dual-task Timed Up and Go, ESM= max excursions, ET= elastic taping, FAB= frontal assessment battery, FES-I= Falls Efficacy Scale International, FGA= functional gate assessment, FGS= fast gait speed, FRT= functional reach test, FTSTS= Five Times Sit-to-Stand Test, FOG-Q= Freezing of Gait Questionnaire, FWD= forward, GAI= Geriatric Anxiety Inventory, GDS= Geriatric Depression Scale, GFI= gait fatigue index, HADS= Hamilton Anxiety and Depression Scale, HWS= Holistic Well-being Scale, HY= Hoehn & Yahr stage, ICPH= Inner Correspondence and feelings of Peaceful Relief, IPAQ= International Physical Activity Questionnaire, LOS= Limits of stability, LRI= locomotor rehabilitation index, MDS-UPDRS= Movement Disorders Society Unified Parkinson Disease Rating Scale, Mini-BESTest= Mini Balance Evaluation Systems Test, MoCA= Montreal Cognitive Assessment, MMSE= Mini-Mental State Examination, MPAS= Modified Parkinson’s Activity Scale, MRT= Mental Rotation Task, ms= milliseconds, NA= not applicable, NFOG-Q= New Freezing of Gait Questionnaire, NIH= National Institutes of Health, NMS= Non-motor Symptom scale, NMSQ= Parkinson’s Disease Non-Motor Symptom Questionnaire, NW= nordic walking, PAS= Parkinson’s Activity Scale, PD= Parkinson’s Disease, PDQ-8= Parkinson’s Disease Questionnaire 8, PDQ-39= Parkinson’s Disease Questionnaire 39, PDQL= Parkinson’s disease Quality of Life questionnaire, PDSS-2= Parkinson's Disease Sleep Scale-2, PFS-16= Parkinson's Disease Fatigue Scale, POMA= Performance-Oriented Mobility Assessment, PPT= Purdue pegboard test, PWT= power training, SAS= Starkstein Apathy Scale, SDS= Self-rating Depression Scale, sec= seconds, SF-36= Short Form (36) Health Survey, SLS= single leg stance, SS180= standing-start 180° turn test, SRT= Sit-and-Reach Test, SSW= Self-selected Walking Speed, STAI= State-Trait Anxiety Inventory, TIS= Trunk Impairment Scale, TUG= Timed Up and Go, TMT= Trail Making Test, UPCRS= Unified Parkinson's Comprehensive Rating Scale, VAS= Visual Analog Scale, VPS= Vitality Plus Scale, WQ= Wuqinxi Qigong.

**References**

1. Ni M, Mooney K, Signorile JF. Controlled pilot study of the effects of power yoga in Parkinson's disease. Complement Ther Med. 2016;25:126-31.

2. Kwok JYY, Kwan JCY, Auyeung M, Mok VCT, Lau CKY, Choi KC, et al. Effects of Mindfulness Yoga vs Stretching and Resistance Training Exercises on Anxiety and Depression for People With Parkinson Disease: A Randomized Clinical Trial. JAMA Neurol. 2019;76(7):755-63.

3. Van Puymbroeck M, Walter AA, Hawkins BL, Sharp JL, Woschkolup K, Urrea-Mendoza E, et al. Functional Improvements in Parkinson's Disease Following a Randomized Trial of Yoga. Evid Based Complement Alternat Med. 2018;2018:8516351.

4. Cherup NP, Strand KL, Lucchi L, Wooten SV, Luca C, Signorile JF. Yoga Meditation Enhances Proprioception and Balance in Individuals Diagnosed With Parkinson's Disease. Percept Mot Skills. 2021;128(1):304-23.

5. Walter AA, Adams EV, Van Puymbroeck M, Crowe BM, Urrea-Mendoza E, Hawkins BL, et al. Changes in Nonmotor Symptoms Following an 8-Week Yoga Intervention for People with Parkinson's Disease. Int J Yoga Therap. 2019;29(1):91-9.

6. Kwok JYY, Choi EPH, Lee JJ, Lok KYW, Kwan JCY, Mok VCT, et al. Effects of Mindfulness Yoga Versus Conventional Physical Exercises on Symptom Experiences and Health-related Quality of Life in People with Parkinson's Disease: The Potential Mediating Roles of Anxiety and Depression. Ann Behav Med. 2022;56(10):1068-81.

7. Elangovan N, Cheung C, Mahnan A, Wyman JF, Tuite P, Konczak J. Hatha yoga training improves standing balance but not gait in Parkinson's disease. Sports Med Health Sci. 2020;2(2):80-8.

8. Ni M, Signorile JF, Mooney K, Balachandran A, Potiaumpai M, Luca C, et al. Comparative Effect of Power Training and High-Speed Yoga on Motor Function in Older Patients With Parkinson Disease. Arch Phys Med Rehabil. 2016;97(3):345-54.e15.

9. Wang T, Xiao G, Li Z, Jie K, Shen M, Jiang Y, et al. Wuqinxi Exercise Improves Hand Dexterity in Patients with Parkinson's Disease. Evid Based Complement Alternat Med. 2020;2020:8352176.

10. Shen M, Pi YL, Li Z, Song T, Jie K, Wang T, et al. The Feasibility and Positive Effects of Wuqinxi Exercise on the Cognitive and Motor Functions of Patients with Parkinson's Disease: A Pilot Study. Evid Based Complement Alternat Med. 2021;2021:8833736.

11. Wan Z, Liu X, Yang H, Li F, Yu L, Li L, et al. Effects of Health Qigong Exercises on Physical Function on Patients with Parkinson's Disease. J Multidiscip Healthc. 2021;14:941-50.

12. Xiao CM, Zhuang YC. Effect of health Baduanjin Qigong for mild to moderate Parkinson's disease. Geriatr Gerontol Int. 2016;16(8):911-9.

13. Liu XL, Chen S, Wang Y. Effects of Health Qigong Exercises on Relieving Symptoms of Parkinson's Disease. Evid Based Complement Alternat Med. 2016;2016:5935782.

14. Moon S, Sarmento CVM, Steinbacher M, Smirnova IV, Colgrove Y, Lai SM, et al. Can Qigong improve non-motor symptoms in people with Parkinson's disease - A pilot randomized controlled trial? Complement Ther Clin Pract. 2020;39:101169.

15. Li Z, Wang T, Shen M, Song T, He J, Guo W, et al. Comparison of Wuqinxi Qigong with Stretching on Single- and Dual-Task Gait, Motor Symptoms and Quality of Life in Parkinson's Disease: A Preliminary Randomized Control Study. Int J Environ Res Public Health. 2022;19(13).

16. Wang Z, Pi Y, Tan X, Wang Z, Chen R, Liu Y, et al. Effects of Wu Qin Xi exercise on reactive inhibition in Parkinson's disease: A randomized controlled clinical trial. Front Aging Neurosci. 2022;14:961938.

17. Amano S, Nocera JR, Vallabhajosula S, Juncos JL, Gregor RJ, Waddell DE, et al. The effect of Tai Chi exercise on gait initiation and gait performance in persons with Parkinson's disease. Parkinsonism Relat Disord. 2013;19(11):955-60.

18. Li F, Harmer P, Liu Y, Eckstrom E, Fitzgerald K, Stock R, et al. A randomized controlled trial of patient-reported outcomes with tai chi exercise in Parkinson's disease. Mov Disord. 2014;29(4):539-45.

19. Gao Q, Leung A, Yang Y, Wei Q, Guan M, Jia C, et al. Effects of Tai Chi on balance and fall prevention in Parkinson's disease: a randomized controlled trial. Clin Rehabil. 2014;28(8):748-53.

20. Kurt EE, Büyükturan B, Büyükturan Ö, Erdem HR, Tuncay F. Effects of Ai Chi on balance, quality of life, functional mobility, and motor impairment in patients with Parkinson's disease<sup/>. Disabil Rehabil. 2018;40(7):791-7.

21. Pérez de la Cruz S. Effectiveness of aquatic therapy for the control of pain and increased functionality in people with Parkinson's disease: a randomized clinical trial. Eur J Phys Rehabil Med. 2017;53(6):825-32.

22. Pérez-de la Cruz S. A bicentric controlled study on the effects of aquatic Ai Chi in Parkinson disease. Complement Ther Med. 2018;36:147-53.

23. Pérez-de la Cruz S. Mental health in Parkinson's disease after receiving aquatic therapy: a clinical trial. Acta Neurol Belg. 2019;119(2):193-200.

24. Khuzema A, Brammatha A, Arul Selvan V. Effect of home-based Tai Chi, Yoga or conventional balance exercise on functional balance and mobility among persons with idiopathic Parkinson's disease: An experimental study. Hong Kong Physiother J. 2020;40(1):39-49.

25. Zhang TY, Hu Y, Nie ZY, Jin RX, Chen F, Guan Q, et al. Effects of Tai Chi and Multimodal Exercise Training on Movement and Balance Function in Mild to Moderate Idiopathic Parkinson Disease. Am J Phys Med Rehabil. 2015;94(10 Suppl 1):921-9.

26. Poier D, Rodrigues Recchia D, Ostermann T, Büssing A. A Randomized Controlled Trial to Investigate the Impact of Tango Argentino versus Tai Chi on Quality of Life in Patients with Parkinson Disease: A Short Report. Complement Med Res. 2019;26(6):398-403.

27. Duncan RP, Earhart GM. Are the effects of community-based dance on Parkinson disease severity, balance, and functional mobility reduced with time? A 2-year prospective pilot study. J Altern Complement Med. 2014;20(10):757-63.

28. Rios Romenets S, Anang J, Fereshtehnejad SM, Pelletier A, Postuma R. Tango for treatment of motor and non-motor manifestations in Parkinson's disease: a randomized control study. Complement Ther Med. 2015;23(2):175-84.

29. Hashimoto H, Takabatake S, Miyaguchi H, Nakanishi H, Naitou Y. Effects of dance on motor functions, cognitive functions, and mental symptoms of Parkinson's disease: a quasi-randomized pilot trial. Complement Ther Med. 2015;23(2):210-9.

30. Shanahan J, Morris ME, Bhriain ON, Volpe D, Lynch T, Clifford AM. Dancing for Parkinson Disease: A Randomized Trial of Irish Set Dancing Compared With Usual Care. Arch Phys Med Rehabil. 2017;98(9):1744-51.

31. Hulbert S, Ashburn A, Roberts L, Verheyden G. Dance for Parkinson's-The effects on whole body co-ordination during turning around. Complement Ther Med. 2017;32:91-7.

32. Lee HJ, Kim SY, Chae Y, Kim MY, Yin C, Jung WS, et al. Turo (Qi Dance) Program for Parkinson's Disease Patients: Randomized, Assessor Blind, Waiting-List Control, Partial Crossover Study. Explore (NY). 2018;14(3):216-23.

33. Michels K, Dubaz O, Hornthal E, Bega D. "Dance Therapy" as a psychotherapeutic movement intervention in Parkinson's disease. Complement Ther Med. 2018;40:248-52.

34. Rawson KS, McNeely ME, Duncan RP, Pickett KA, Perlmutter JS, Earhart GM. Exercise and Parkinson Disease: Comparing Tango, Treadmill, and Stretching. J Neurol Phys Ther. 2019;43(1):26-32.

35. Kalyani HHN, Sullivan KA, Moyle G, Brauer S, Jeffrey ER, Kerr GK. Impacts of dance on cognition, psychological symptoms and quality of life in Parkinson's disease. NeuroRehabilitation. 2019;45(2):273-83.

36. Tillmann AC, Swarowsky A, Corrêa CL, Andrade A, Moratelli J, Boing L, et al. Feasibility of a Brazilian samba protocol for patients with Parkinson's disease: a clinical non-randomized study. Arq Neuropsiquiatr. 2020;78(1):13-20.

37. Frisaldi E, Bottino P, Fabbri M, Trucco M, De Ceglia A, Esposito N, et al. Effectiveness of a dance-physiotherapy combined intervention in Parkinson's disease: a randomized controlled pilot trial. Neurol Sci. 2021;42(12):5045-53.

38. Foster ER, Golden L, Duncan RP, Earhart GM. Community-based Argentine tango dance program is associated with increased activity participation among individuals with Parkinson's disease. Arch Phys Med Rehabil. 2013;94(2):240-9.

39. Kunkel D, Fitton C, Roberts L, Pickering RM, Roberts HC, Wiles R, et al. A randomized controlled feasibility trial exploring partnered ballroom dancing for people with Parkinson's disease. Clin Rehabil. 2017;31(10):1340-50.

40. Solla P, Cugusi L, Bertoli M, Cereatti A, Della Croce U, Pani D, et al. Sardinian Folk Dance for Individuals with Parkinson's Disease: A Randomized Controlled Pilot Trial. J Altern Complement Med. 2019;25(3):305-16.

41. Li F, Wang D, Ba X, Liu Z, Zhang M. The comparative effects of exercise type on motor function of patients with Parkinson's disease: A three-arm randomized trial. Front Hum Neurosci. 2022;16:1033289.

42. Maciel DP, Mesquita VL, Marinho AR, Ferreira GM, Abdon AP, Maia FM. Pilates method improves balance control in Parkinson's disease patients: An open-label clinical trial. Parkinsonism Relat Disord. 2020;77:18-9.

43. Mollinedo-Cardalda I, Cancela-Carral JM, Vila-Suárez MH. Effect of a Mat Pilates Program with TheraBand on Dynamic Balance in Patients with Parkinson's Disease: Feasibility Study and Randomized Controlled Trial. Rejuvenation Res. 2018;21(5):423-30.

44. Göz E, Çolakoğlu BD, Çakmur R, Balci B. Effects of Pilates and Elastic Taping on Balance and Postural Control in Early Stage Parkinson's Disease Patients: A Pilot Randomised Controlled Trial. Noro Psikiyatr Ars. 2021;58(4):308-13.

45. Cugusi L, Solla P, Serpe R, Carzedda T, Piras L, Oggianu M, et al. Effects of a Nordic Walking program on motor and non-motor symptoms, functional performance and body composition in patients with Parkinson's disease. NeuroRehabilitation. 2015;37(2):245-54.

46. Monteiro EP, Franzoni LT, Cubillos DM, de Oliveira Fagundes A, Carvalho AR, Oliveira HB, et al. Effects of Nordic walking training on functional parameters in Parkinson's disease: a randomized controlled clinical trial. Scand J Med Sci Sports. 2017;27(3):351-8.

47. Bang DH, Shin WS. Effects of an intensive Nordic walking intervention on the balance function and walking ability of individuals with Parkinson's disease: a randomized controlled pilot trial. Aging Clin Exp Res. 2017;29(5):993-9.

48. Granziera S, Alessandri A, Lazzaro A, Zara D, Scarpa A. Nordic Walking and Walking in Parkinson's disease: a randomized single-blind controlled trial. Aging Clin Exp Res. 2021;33(4):965-71.

49. Mak MKY, Wong-Yu ISK. Six-Month Community-Based Brisk Walking and Balance Exercise Alleviates Motor Symptoms and Promotes Functions in People with Parkinson's Disease: A Randomized Controlled Trial. J Parkinsons Dis. 2021;11(3):1431-41.

50. Szefler-Derela J, Arkuszewski M, Knapik A, Wasiuk-Zowada D, Gorzkowska A, Krzystanek E. Effectiveness of 6-Week Nordic Walking Training on Functional Performance, Gait Quality, and Quality of Life in Parkinson's Disease. Medicina (Kaunas). 2020;56(7).

51. Franzoni LT, Monteiro EP, Oliveira HB, da Rosa RG, Costa RR, Rieder C, et al. A 9-Week Nordic and Free Walking Improve Postural Balance in Parkinson's Disease. Sports Med Int Open. 2018;2(2):E28-e34.

52. Sangarapillai K, Norman BM, Almeida QJ. BOX vs Sensory Exercise for Parkinson's Disease: A Double-Blinded Randomized Controlled Trial. Neurorehabil Neural Repair. 2021;35(9):769-77.

53. Domingos J, de Lima ALS, Steenbakkers-van der Pol T, Godinho C, Bloem BR, de Vries NM. BOX with and without Kicking Techniques for People with Parkinson's Disease: An Explorative Pilot Randomized Controlled Trial. J Parkinsons Dis. 2022;12(8):2585-93.

54. Combs SA, Diehl MD, Chrzastowski C, Didrick N, McCoin B, Mox N, et al. Community-based group exercise for persons with Parkinson disease: a randomized controlled trial. NeuroRehabilitation. 2013;32(1):117-24.

55. Langer A, Hasenauer S, Flotz A, Gassner L, Pokan R, Dabnichki P, et al. A randomised controlled trial on effectiveness and feasibility of sport climbing in Parkinson's disease. NPJ Parkinsons Dis. 2021;7(1):49.

56. Shujaat F, Soomro N, Khan M. The effectiveness of Kayaking exercises as compared to general mobility exercises in reducing axial rigidity and improve bed mobility in early to mid stage of Parkinson's disease. Pak J Med Sci. 2014;30(5):1094-8.
